# Integrated survival analysis using an event-time approach in a Bayesian framework

**DOI:** 10.1002/ece3.1399

**Published:** 2015-01-17

**Authors:** Daniel P Walsh, Victoria J Dreitz, Dennis M Heisey

**Affiliations:** 1 National Wildlife Health Center, United States Geological Survey6006 Schroeder Road, Madison, Wisconsin, 53711; 2 Wildlife Biology Program and Avian Science Center, College of Forestry and Conservation, University of MontanaMontana, 59812

**Keywords:** *Charadrius montanus*, continuous time, detection probability, event time, hazard rate, mountain plover, simulation, survival, unknown fate

## Abstract

Event-time or continuous-time statistical approaches have been applied throughout the biostatistical literature and have led to numerous scientific advances. However, these techniques have traditionally relied on knowing failure times. This has limited application of these analyses, particularly, within the ecological field where fates of marked animals may be unknown. To address these limitations, we developed an integrated approach within a Bayesian framework to estimate hazard rates in the face of unknown fates. We combine failure/survival times from individuals whose fates are known and times of which are interval-censored with information from those whose fates are unknown, and model the process of detecting animals with unknown fates. This provides the foundation for our integrated model and permits necessary parameter estimation. We provide the Bayesian model, its derivation, and use simulation techniques to investigate the properties and performance of our approach under several scenarios. Lastly, we apply our estimation technique using a piece-wise constant hazard function to investigate the effects of year, age, chick size and sex, sex of the tending adult, and nesting habitat on mortality hazard rates of the endangered mountain plover (*Charadrius montanus*) chicks. Traditional models were inappropriate for this analysis because fates of some individual chicks were unknown due to failed radio transmitters. Simulations revealed biases of posterior mean estimates were minimal (≤ 4.95%), and posterior distributions behaved as expected with RMSE of the estimates decreasing as sample sizes, detection probability, and survival increased. We determined mortality hazard rates for plover chicks were highest at <5 days old and were lower for chicks with larger birth weights and/or whose nest was within agricultural habitats. Based on its performance, our approach greatly expands the range of problems for which event-time analyses can be used by eliminating the need for having completely known fate data.

## Introduction

Integrated analyses combine information from various data sources to make efficient use of multiple and varied forms of information while properly accounting for covariances of estimated parameters. This permits a more thorough examination of the process and observation models of interest (Nasution et al. 2001; Brooks et al. 2004). Additionally, the results of these analyses provide more robust parameter estimates by incorporating all available information compared to conducting individual analyses for each data type and combining estimates in some *ad hoc* manner (Nasution et al. 2001; Brooks et al. 2004). Although not new, these techniques have become increasingly popular and have been employed in survival analyses using capture–recapture methods where band recovery and live recapture data have been jointly analyzed to improve estimates of survival (Burnham 1993; Catchpole et al. 1998; Barker et al. 2005). Additionally, Nasution et al. (2001) developed survival estimates through a joint analysis of resighting and radiotelemetry capture–recapture data based on discrete approaches (Pollock et al. 1989b).

Event-time or continuous-time survival analyses have a rich history in human biomedical investigations where they have been widely applied and have led to numerous advances within the field (Lee and Wang 2003; Heisey et al. 2007; Heisey 2009). However, their use beyond this arena has generally been limited (Heisey et al. 2007). For example, in ecology, event-time analyses have seen some application, although it has been mainly confined to studies where the time to failure/death is known with certainty or can be assigned to some temporal interval (e.g., nest survival, force of infection, maturation, and survival of radio-collared animals) (Heisey et al. 2006, 2010; Cao et al. 2009; Ergon et al. 2009; Conn et al. 2012; Halstead et al. 2012). This narrow application largely arises from differences in data type and structure collected during wildlife investigations compared to biomedical studies. Continuous-time approaches have also seen limited application in modeling the capture/detection process in capture–recapture and spatial capture–recapture models used to estimate population sizes and densities, respectively (Yip et al. 1996; Hwang and Chao 2002; Farcomeni and Scacciatelli 2013; Borchers et al. 2014). They are particularly useful when the detector operates continuously (e.g., remote camera). Given the power of these techniques and their ability to model both the data collection and underlying biological processes in a statistically rigorous manner, while accounting for the dynamic unfolding nature of these processes in time, the creation of methods that permit expanded use of these statistical analytical tools for a greater range of wildlife investigations is desirable.

To that end, we have developed a new statistical method that expands the framework of event-time analyses and makes it more widely applicable to studies where individual fates may be unknown. In particular, we provide an integrated event-time analysis that combines information from individuals whose failure time can be ascertained exactly or minimally is known to an interval (e.g., radio-marked animals) with individuals whose outcome is unknown (e.g., marked animals). The modeling of a detection process provides the backbone for formulating this integrated model and allowing estimation of the parameters of interest. We provide the statistical model, its derivation, and use simulation techniques to investigate the performance of this model under a variety of scenarios. It is worth noting that although we formulate the model, simulations, and case study in terms of mortality hazard rates, the model can be applied to other types of hazard rates (e.g., infection) where unknown fates occur within the dataset and a detection process can be modeled.

Lastly, we apply the technique to estimate survival of mountain plover chicks (*Charadrius montanus*) in Colorado, USA, where fates of individual chicks may be unknown due to intermittent or failed radio transmitters. The mountain plover is an endangered, upland shorebird that has experienced steep, constant declines in population size across its range since 1966. Factors driving population declines appear to be acting on reproductive output of the species (Knopf and Wunder 2006), including chick survival (Dinsmore et al. 2010); however, demographic information, including estimates of vital rates for birds transitioning through the chick stage (i.e., the period from hatching to fledging), is lacking. Standard event-time analyses (Heisey 2009) are not appropriate for this system because the radio transmitters often could not be detected during the observation period because of weak signals or transmitter malfunctions. Thus, this system is a perfect candidate on which to employ our analysis technique with chicks with known fates playing the role of radio-marked animals and the remaining chicks corresponding to marked individuals in the above description.

### Model development

#### Assumptions and data needs

When assessing individual survival, biologists typically attempt to capture a random sample of individuals from the population(s) of interest and apply some type of mark to captured animals, which may or may not be individually identifiable. Animals are then recaptured or observed at some interval, and survival estimates are derived from this information. For the current study, we will assume individuals are captured and uniquely marked using either a radio collar or a unique mark, although the latter can be any individually identifiable mark, or as in our case study, a malfunctioning radio transmitter. But for simplicity, we will describe these simply as marks throughout the following discussion. Our model assumes the population is geographically closed, radio-collared animals are checked periodically for mortality, and individuals with functioning collars have a detection probability of one. For animals receiving a mark, we will assume that, once marked, the study area is surveyed at intervals for these individuals throughout the length of the study. Thus, detection probability is less than one for marked animals. A reasonable survey protocol may be to survey the study area for marked individuals while conducting mortality checks of radio-collared animals; however, this is not required.

For our event-time analyses, three types of information are needed as follows: (1) the date each individual (e_*i*_) was captured or marked; (2) the date the individual was last-known alive (*r*_*i*_); and (3) the first date the individual was known to have died/failed or was censored (*s*_*i*_; Heisey et al. 2007). We assume that the event time (*T*_*i*_) is only known to the interval [*r*_*i*_, *s*_*i*_]. In general, most marked animals will be considered to be right-censored (i.e., *s*_*i*_ = ∞) owing to the low probability of discovering dead marked animals. Our model also requires enumeration of either observation times of marked animals or whether an individual was observed during each survey occasion depending on whether the detection process is modeled as a continuous or discrete process. Additionally, any pertinent covariate information may be collected.

#### Data likelihood

We decompose the likelihood function into two components. The first component represents the information contributed by radio-collared animals, and the survival components have been previously described by Heisey et al. (2007) and have the following form:

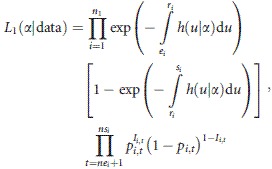
1

where *n*_1_ = number of radio-collared animals, *h(u) *= the hazard function, ***α ***= parameters associated with *h*(*u*), *p*_*i,t *_= probability of detection for the *i*th radio-collared animal during the *t*th observation survey, *I*_*i,t*_* *= an indicator of successful observation of the *i*th radio-collared animal during the *t*th observation survey, *ne*_*i*_* *= the number of observation surveys prior to *e*_*i*_ for the *i*th radio-collared animal, ns_*i *_= the last observation survey the *i*th radio-collared animal was observed, and other variables are as described previously.

This construction allows for both interval and right censoring and accounts for staggered entry or left truncation of individuals into the marked samples (Pollock et al. 1989a,b). The hazard function can take any form, and common choices include constant, log-logistic, Weibull, and the piece-wise constant hazards. The final term in the likelihood is the product of a series of Bernoulli random variables and evaluates the number of observations of the *i*th marked animal during the interval from the date of marking to the date of the last survey which it was observed. It is noteworthy that the number of surveys that an individual is available for observation is individual-specific conditional on *e*_*i*_, unless observation surveys begin after all individuals are marked, which implies *ne*_*i *_= 0 for all individuals. We chose to model each individual’s encounter histories as outcomes of Bernoulli trials from the origin/study start until the last survey occasion at which each marked animal was observed; however, other models can be used including continuous detection process models (Yip et al. 1996; Hwang and Chao 2002). Following a reviewer suggestion, we have included the last term in the likelihood for radio-collared animals solely for generality, and its inclusion requires the assumption that observers searched for radio-collared animals in the same manner and with the same effort that they searched for marked animals, and did not employ the use of the radio transmitter to observe the animal. Inclusion of this function also assumes detection probability is independent of the type of mark deployed on the animal. If this assumption holds, including this term in *L*_1_ will increase the precision of estimated posterior distributions; however, we believe this assumption does not hold in general because radio transmitters permit the consistent location of radio-collared individuals. In our experience, this decreases the search effort and increases the detection probability of radio-collared individuals because over time observers learn where to search for and expect to find these animals. Therefore, in practice, we would normally remove the last term from *L*_1_, and we removed it during the subsequent analyses performed during the simulations and case study.

The second component represents the contributions to the likelihood from marked animals. To account for the fact that the detection probability for this group is almost assuredly less than one, we model the detection process, which also provides information regarding the random variable, death/event time. Thus, the second likelihood component is as follows:

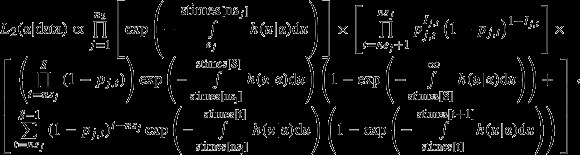
2where *n*_2 _= number of marked animals, *S *= number of observation surveys, stimes = vector of observation survey times, and other variables are as described previously. There are three distinct multiplicative elements (in brackets) in equation 2. The first element is similar to the first likelihood component and models survival, while the individual is known alive. The second element is the product of Bernoulli trials and models the detection process during observation surveys as previously described. The final element accounts for unknown fates of marked animals past *ns*_*j*_: the probability they survived throughout the study and were never observed or died between *ns*_*j*_ and the study’s end. Death can be thought of as a competing risk which censors the future recapturing observations, which can be described as latent observations. Because all possible future recapture histories are censored, the sum of their postdeath conditioned probabilities is one and would contribute a constant term to the likelihood, so such hypothetical histories need not be explicitly included.

We construct the joint likelihood for radio-collared and marked animals by assuming independent death times for each individual. The resulting joint likelihood is as follows: *L = L*_1 _*× L*_2_, and because the two components share hazard parameters, this represents an integrated analysis approach. Covariates can be easily added to the likelihood using a proportional hazards approach and through standard link functions for the detection probability (Heisey et al. 2007).

#### Priors and posterior

The joint posterior for the parameters (***α***, *p*_*j,t*_) is proportional to the product of the joint likelihood (*L*) and prior distributions for the unknown parameters. In the simulation study and analysis of mountain plover data described below, we assume probability of detection is constant across observation surveys. Therefore, we use the following in place of the product of Bernoulli trials in *L*_2_:

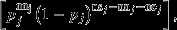
3

where *nn*_*j*_ = the number of sightings of the *i*th marked animal. We then specify a Bayes–Laplace uniform (0,1) prior for the *p*_*j*_. We also specify similarly weak priors for ***α*** parameters; however, a wide range of priors may be specified to provide the hazard function posited structure a priori based on knowledge gathered from previous studies or expert opinion (Gelman et al. 2004).

For point and interval parameter estimation, we sample from the joint posterior distribution of ***α*** and *p*_*j*_ using Markov chain Monte Carlo (MCMC) algorithms to obtain posterior means and credible intervals (CIs). Specifically, we use the Hit and Run Metropolis (HARM; Gilks and Roberts, 1996) in the Laplace’s Demon package in Program R (Statisticat LLC 2012; R Development Core Team 2013).

### Model application

#### Simulation studies

We conducted a simulation study to examine the performance of our model. We chose a piece-wise constant mortality hazard model comprised of two different hazard values for the simulations. This model is a reasonable model for species with high survival throughout most of the year but increased hazard during some season (e.g., hunting season). Our simulation examples were based on weekly surveys for marked individuals and mortality checks of radio-collared animals.

We generated data for several different combinations of annual survival and detection probabilities. These combinations we deemed plausible for typical field studies. We used annual survival probabilities of 0.25, 0.55, and 0.85 and individual detection probabilities of 0.20, 0.40, and 0.90 for marked animals at each survey occasion. For each simulation, we chose a unique combination of annual survival probability and detection probability. We specified the hazard ratio of hunting to nonhunting seasons as two and set the hunting season to occur from 10/1 through 12/31 each year. For most combinations of survival and detection probabilities, we set the survey period to be 477 days in length; however, if annual survival was 0.85, the survey period was 1569 days long to assure an adequate number of deaths for estimation. We examined two different sample sizes of marked individuals – the first was 40 radio-collared animals and 40 marked animals, which is a reasonable sample size for many survival studies. The second was 250 radio-collared animals and 250 marked animals, representing an exceptionally large sample size for wildlife studies. We randomly assigned the time of capture and marking of each individual to fall within the interval of day [1, 30] of the study. We randomly generated death times for each individual using a two-step process. First, using a random draw from a multinomial distribution, we assigned which interval of the piece-wise constant hazard function death occurred where individual multinomial probabilities were geometrically distributed. The number of parameters (*m*) of the distribution equaled the number of change points in the piece-wise constant hazard function plus one. The individual parameters for *k = *2 to *m−*1 were as follows:

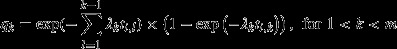
4

where *λ*_*k*_ = is the hazard rate during the *k*th interval of the piece-wise constant hazard function, *t*_*i,k*_ = the length of the *k*th interval for the *i*th individual, and *q*_*0*_* = *1. For *k *=* *1, the parameter was simply the probability of failing during the first interval, while for *k *= m, the probability was one minus the sum of *m−*1 other probabilities. Each individual had a unique vector of parameters because of the staggered entry of individuals into the marked sample. Once we generated the interval, we randomly generated the failure time within the interval from truncated exponential distribution with rate parameter (*λ*_*k*_). The piece-wise exponentially distributed death time was then calculated as the sum of the length of intervals prior to the selected interval and the generated failure time. If an individual’s time of death exceeded the study length, we treated it as right-censored. Lastly, we generated last-known alive and first-known dead times of marked animals given these individual death times. We surveyed marked animals weekly throughout the study; thus, for radio-collared animals, we specified *r*_*i*_ as the lower bound of the survey interval containing the death time and *s*_*i*_ as the upper bound of the same interval. If the individual was right-censored, ns_*i*_ was the last survey time and *s*_*i*_* *= ∞. For marked animals, we generated whether it was observed during each survey occasion using a Bernoulli distribution with parameter (*p*_*j*_) given that it was alive to be observed. Therefore, *ns*_*j*_ was the last survey during which the animal was observed. We treated all marked animals as right-censored because no death times were recorded for them.

We analyzed the generated data using the Bayesian model and MCMC algorithms previously described. To account for sampling variability associated with the generated entry, failure, and observation times, we ran 500 individual chains for 50,000 iterations each and randomly generated dispersed starting values for parameters from uniform distributions. To assess model performance, we calculated the percent bias (PBS) and root mean square error (RMSE) for the three parameters of interest: the nonhunting mortality hazard rate (*λ*_1_), the hunting mortality hazard rate (*λ*_2_), and the detection probability (*P*), using the means of the posterior distributions for each parameter from each chain. To assess stationarity of each chain, we used the Geweke diagnostic test as applied within the R package, Laplace’s Demon (Geweke 1992; Statisticat LLC 2012).

We performed a second simulation study to examine the effects of varying the number of marked and radio-collared animals in a marked sample of 80 animals. We used an annual survival rate of 0.55 and examined detection probabilities of 0.20 and 0.40. The simulation technique followed the methodology described for the earlier simulation study and examined the same parameters and metrics.

#### Case study: mountain plover chick survival

**Figure 1:**
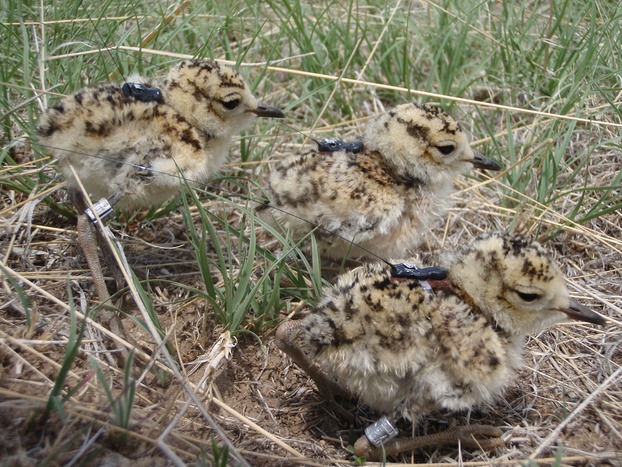
Mountain plover chicks (*Charadrius montanus*) fitted with radio transmitters and metal bands for survival estimation (Photograph credit: Colorado Parks and Wildlife).

To illustrate the use of our technique, we analyzed survival of mountain plover chicks captured and fitted with radio transmitters (Blackburn Transmitters; Pip Lotek Wireless Inc., Canada) at 1 day of age in Colorado, USA, and were observed until they were 30 days old from 2010 to 2012. Thus, we considered an observation survey to be each day a chick was checked for mortality or an observation was attempted. We attached 0.35-g transmitters on chicks at hatching (∽10 g). Transmitters were attached using a modified design of Rappole and Tipton leg harness attachment method (Dreitz et al. 2011). Figure 1 displays mountain plover chicks recently fitted with transmitters. We replaced transmitters at ∽16 days to maintain monitoring until ≥30 days of age. We used the radio transmitters to determine the live/dead status of each chick either through direct observation or changes in location of the radio signal indicating movement. If a radio transmitter failed, the chick was searched for intensively within a 2 km radius of its last-known location. If a chick was discovered, we made daily observation attempts during which their live, dead, or nondetected status was noted. Chicks with failed radio transmitters that were not initially discovered were searched for throughout the study area during each observation survey until it was observed or the study ended.

We measured several covariates that may impact the age-specific mortality hazard rates: chick size at marking measured via mass and tarsus length of chicks, nesting habitat type, year of the study, sex of the chick (male = 1), and sex of the attending adult (male = 1). Habitat types were classified as grassland (reference state), prairie dog colony, agricultural fields, or unknown (i.e., the habitat type of the chick’s nest site could not be determined exactly because it was not in a nest). The effect of year was included because chick survival for most avian species exhibits high annual variation using 2010 as our reference state. We also included tending adult sex because mountain plovers’ breeding system is uniparental which means only one adult tends to the nest and brood (Knopf and Wunder 2006).

We used the likelihood *L* described above for our analysis with modifications to account for the data structure. The time scale on which the hazard is expressed was 1 day. Chicks, whose radio transmitter functioned until they were >30 days of age, were modeled using *L*_1_. However, as previously mentioned, we did not include probability of detection in *L*_1_ because we believed the detection probability for chicks with functioning radio transmitters was higher than those with malfunctioning transmitters. To model the survival and detection process for chicks, whose transmitters failed, we used *L*_2._ The entry age and age for which the chick was last-known alive was used to parameterize the survival element of *L*_2_. The detection probability element of *L*_2_ was estimated using information regarding the number of times the chick was observed and the number of observation surveys after the first survey it was discovered the radio transmitter had failed. The remainder of *L*_2_ was estimated as previously described. We used a log-linear proportional hazards model to incorporate the necessary covariates into the hazard function; with this parameterization, the exponentiated coefficients are interpretable as hazard ratios. We standardized (mean 0, standard deviation 1) each of the continuous covariates prior to analysis. The associated hazard ratios measure relative change in hazard for a one-standard-deviation increase in each covariate value. For the intercept and each of the covariate parameters, we used weak uniform [−5, 5] priors. Two covariates, chick sex (*n *=* *44) and adult sex (*n *=* *72), had missing values. We specified Bernoulli priors for these two covariates and placed Gaussian (*μ *= 0.5, *σ *= 0.05) priors on the parameters of these Bernoulli distributions, which regulates the probability of being male for either covariate to being contained within 45–55% or with a 95% probability. Our informative priors on these parameters reflected our prior belief that probability of chick and tending adult sex being male or female in each case should be roughly equal. We treated *p*_*j*_, the probability of detecting a chick after the radio failure, as a constant.

Because we wanted to investigate whether survival was lower during the first days of life as posited based on field observations, we used a Bayesian hierarchical model and included an age effect (*age*_*k*_) as a random effect for each of *k = *1 to 29 age intervals in our proportional hazards specification. These effects were modeled using a random walk prior with **age** ∽ Gaussian(**0**, **Σ**) to reflect the prior belief that mortality hazard rates for chicks close in age are similar (correlated) and to produce a smoothed hazard curve (Besag et al. 1991; Cressie and Wikle 2011). Following the notation of Cressie and Wikle (2011), we modeled **Σ ** =  (**I-H)**^−1^**M,** where I is a *k  ×  k* identity matrix, **M **= *τ*^2^**I**, *τ*^2^ is the precision, and **H** is the neighborhood matrix with entries *h*_*i,j*_ = 0 if age interval *j* does not precede or follow interval *i,* otherwise, *h*_*i,j*_ = ½, for 1 <  *i *<* *29, and *h*_*i,j*_ = 1 for *i *=* *1 or 29. We specified a weakly informative uniform [0,5] prior on the standard deviation parameter 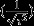. We also analyzed the dataset using a piece-wise constant hazard function with the first 3 age intervals (age < 4 days old) having one mortality hazard rate and the remaining age intervals having a second rate. We specified a weak uniform [−5, 5] for the log hazard parameters in the piece-wise constant model. Lastly, we created a constant proportional hazards model where the mortality hazard rate did not vary with age. We used deviance information criterion (DIC; Spiegelhalter et al. 2002; Gelman et al. 2004) to select the most appropriate of these three candidate models from which to make inference.

For the model with the lowest DIC value, we ran three chains with dispersed starting values for 500,000 repetitions and discarded the first 250,000 as burn-in. We used graphical diagnostics including trace and autocorrelation plots, and the Brooks, Gelman, and Rubin statistics (Brooks and Gelman 1998) to assess convergence via the boa package (Smith 2007) of program R (R Development Core Team 2013). To examine sensitivity of the posterior distributions for each of the hazard covariates and piece-wise constant hazards to our choice of weakly informative prior distributions, we reanalyzed the top model using a central *t*-distribution with 5 degrees of freedom and Gaussian (*μ * =  0, *σ * =  2.24) distribution as priors. We then compared each of the estimated posterior distributions for the piece-wise hazards and covariate effects for discrepancies between each of these prior distributions, which would indicate sensitivity to the choice of priors. We also conducted posterior predictive checks to assess the goodness of fit of this model (Gelman et al. 2014). We examined two test statistics, the overall mean failure age of chicks known to die during the study, and the difference of predicted failure ages from the posterior predictive distribution and observed failure ages for this same group of chicks. We calculated Bayesian *P*-values for these test statistics (Gelman et al. 2014). Lastly, to evaluate the validity of our proportional hazards assumption, we added covariate*age interaction terms for each of the covariates from the top model whose 95% credible intervals [CIs] did not include zero (Lee and Wang 2003). We followed the same analysis procedures described above for our top model and examined the posterior distributions of these interaction terms and determined whether their 95% credible intervals included zero, which would indicate evidence that the proportional hazard assumption was met. However, we did not examine an age*year effect because our random walk hazard model already captured this interaction.

## Results

### Simulation studies

Our MCMC chains for the simulation studies all were evidenced to have converged based on graphical checks and the Geweke diagnostic values. The results of our first simulation study are shown in Table 1, which presents the PBS and RMSE for each of the parameters for the two different sample sizes investigated. The biases in estimated posterior means were minimal with the largest PBS being 4.95% for *λ*_2_ when annual survival was 0.85 and detection probability was 0.40, using sample sizes of 40 marked and 40 radio-collared individuals. Table 1 illustrates that as expected, the RMSE of the estimated posterior means decreased as sample sizes, detection probability, and survival increased. For example, all estimated posterior means had minimal PBS values (i.e., ≤ ±4.2%).

**Table 1:** Simulation results for the parameters (Par): nonhunting hazard (λ_1_), the hunting hazard (λ_2_), and detection probability (*P*) when annual survival, detection probability, and sample size of radio-collared and marked animals were varied

			Mean		Relative bias (%)		RMSE	
Annual survival	Par	True value	Sample 40/40	Sample 250/250	Sample 40/40	Sample 250/250	Sample 40/40	Sample 250/250
0.25	*λ*_1_	0.0030	0.0030	0.0030	−0.094	0.086	0.0006	0.0002
	*λ*_2_	0.00607	0.0061	0.0061	0.163	0.303	0.0010	0.0004
	*P*	0.2000	0.2001	0.2002	0.064	0.089	0.0128	0.0052
0.25	*λ*_1_	0.0030	0.0030	0.0030	−0.254	0.018	0.0005	0.0002
	*λ*_2_	0.00607	0.0061	0.0061	1.149	0.301	0.0009	0.0004
	*P*	0.4000	0.4008	0.4004	0.207	0.108	0.0219	0.0063
0.25	*λ*_1_	0.0030	0.0030	0.0030	−0.015	−0.028	0.0005	0.0002
	*λ*_2_	0.00607	0.0061	0.0061	0.231	0.379	0.0009	0.0004
	*P*	0.9000	0.8997	0.8998	−0.030	−0.017	0.0089	0.0036
0.55	*λ*_1_	0.0013	0.0013	0.0013	−3.181	0.256	0.0003	0.0001
	*λ*_2_	0.00262	0.0027	0.0026	2.613	1.139	0.0005	0.0002
	*P*	0.2000	0.2004	0.2002	0.223	0.123	0.0098	0.0040
0.55	*λ*_1_	0.0013	0.0013	0.0013	−2.683	0.224	0.0003	0.0001
	*λ*_2_	0.00262	0.0027	0.0026	2.061	0.882	0.0005	0.0002
	*P*	0.4000	0.4004	0.4004	0.103	0.090	0.0121	0.0047
0.55	*λ*_1_	0.0013	0.0013	0.0013	−0.487	0.221	0.0003	0.0001
	*λ*_2_	0.00262	0.0027	0.0026	2.722	0.949	0.0005	0.0002
	*P*	0.9000	0.8997	0.8999	−0.039	−0.011	0.0051	0.0028
0.85	*λ*_1_	0.0004	0.0004	0.0004	−0.184	1.247	0.0001	0.0001
	*λ*_2_	0.00071	0.0007	0.0007	4.950	0.385	0.0002	0.0001
	*P*	0.2000	0.2003	0.2001	0.158	0.068	0.0052	0.0033
0.85	*λ*_1_	0.0004	0.0004	0.0004	−0.127	1.238	0.0001	0.0001
	*λ*_2_	0.00071	0.0007	0.0007	4.708	0.158	0.0002	0.0001
	*P*	0.4000	0.4004	0.4002	0.098	0.049	0.0064	0.0041
0.85	*λ*_1_	0.0004	0.0004	0.0004	0.092	1.508	0.0001	0.0001
	*λ*_2_	0.00071	0.0007	0.0007	4.244	0.483	0.0002	0.0001
	*P*	0.9000	0.9000	0.8999	−0.004	−0.010	0.0039	0.0024

**Figure 2:**
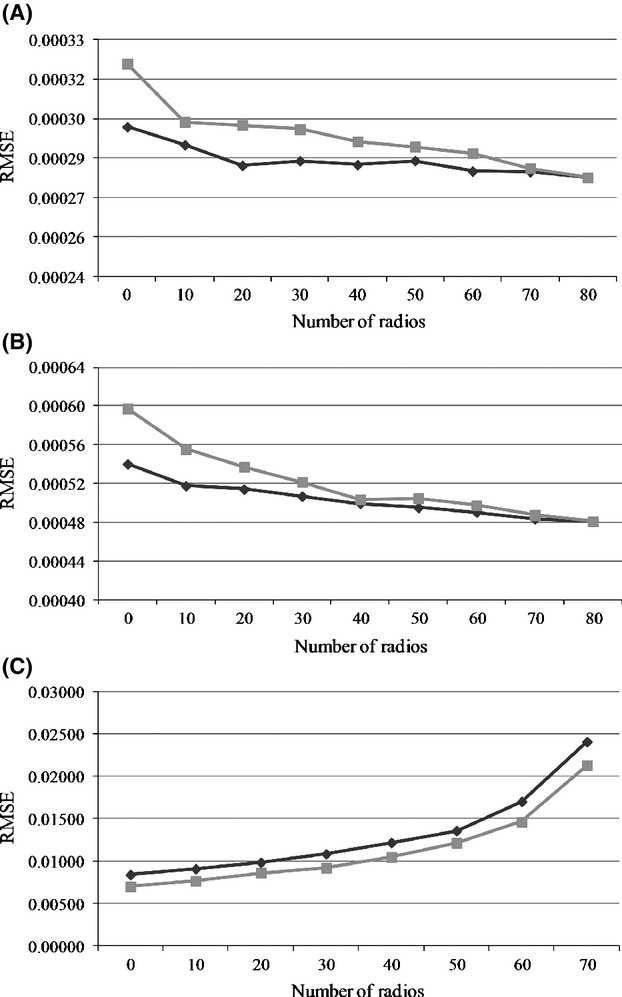
The root mean square error (RMSE) for the nonhunting hazard (*λ*; A), the hunting hazard (*λ*_2_; B), and detection probability (*P*; C) using a detection probability of 0.2 (light gray line) and a detection probability of 0.4 (dark gray line) with varying number of radio-collared animals in the sample.

In our second simulation study, we varied the proportion of radios comprising the sample of individuals. On examination of the RMSE plots in Figure 2 for the parameters, *λ*_1_ and *λ*_2_, we noted the RMSE was highest when there were no radio-collared animals in the sample, and decreased as the number of radio-collared animals increased. The decrease in RMSE values for these parameters with increasing radio-marked animals was more marked when detection probability was specified as 0.20 compared to 0.40. Interestingly, even in the absence of radio-marked animals in the sample, reasonable posterior distributions were still generated. The percent increase in RMSE values between no radio-marked and only radio-marked animals in the sample with a detection probability of 0.40 was 6.9% and 12% for *λ*_1_ and *λ*_2_, respectively, and when detection probability was 0.20, it was 16% and 24% for *λ*_1_ and *λ*_2_, respectively. The PBS values, for detection probability of 0.40 when no radios were included in the sample, were only −2.3% and 2.9%, and for detection probability of 0.20, the PBS values were −4.13% and 4.13% for *λ*_1_ and *λ*_2_, respectively. The RMSE for the nuisance parameter, *p*, behaved differently. It increased as the proportion of radio-collared animals increased.

### Mountain plover chick survival

We captured and marked 234 individual plover chicks during our study. The battery life of the transmitters was ∽ 18 days. As a result, only 91 chicks’ fates were known. Additionally, only 71 chicks were monitored whose radios were detected at every observation prior to death or right censoring at 30 days of age, while the remaining chicks had at least one occasion where they were not detected.

**Figure 3:**
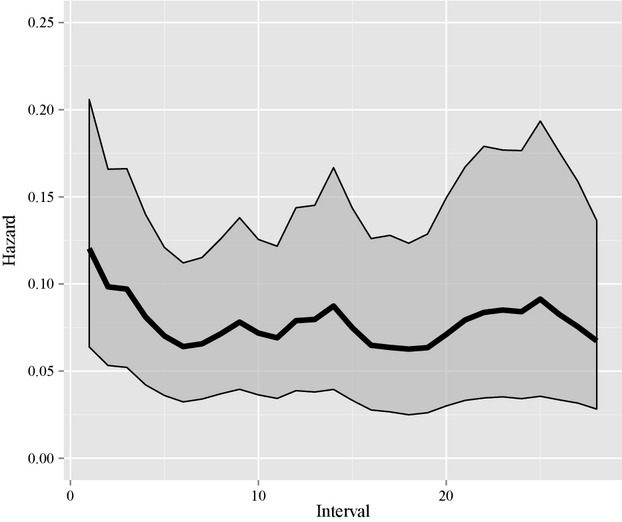
The estimated hazard curve for mountain plover (*Charadrius montanus*) chicks in Colorado, USA, from 2010 to 2012. The dark line represents the mean estimated hazard at each age interval, and the gray envelopes represent 90% credible intervals for the hazard.

Our model selection results demonstrated the piece-wise constant model was best supported by the evidence in the data. The DIC values were 1520.549, 1527.266, and 1543.568 for the piece-wise constant, the random walk model, and the constant hazards model, respectively. Despite having a higher DIC value, we examined the estimated random effect terms from the random walk model. We determined only effects for the first 3 age intervals (i.e., age 1–4 days) had posterior distributions shifted away from zero (i.e., their 90% CIs did not include zero). The estimated hazard curve from this model is shown in Figure 3. Based on these results, we estimated the parameter values and made inference using the piece-wise constant hazard model.

During our analysis using the piece-wise constant model, no evidence of nonconvergence of our chains was observed; the multivariate scale reduction factor was 1.011, and the 0.975 quantiles for the corrected scale reduction factors for all parameters were ≤ 1.018. Graphical examination of the distribution of each of the posterior predictive check test statistics revealed they were mildly left-skewed. Closer examination revealed skewness was the result of underestimating the failure age for a few chicks whose age at death occurred at 30 days. However, based on these test statistics, in general, the model does a reasonable job of fitting the overall mean failure age producing a Bayesian *P*-value of 0.38. It also acceptably fits the individual mean failure ages yielding a Bayesian *P*-value of 0.57.

We did not measure substantial sensitivity to our choice of weakly informative priors. Each of the priors generally yielded similar posterior distributions for each of the parameters and yielded similar inference. The only difference we noted was when using the Gaussian prior, the 95% CIs for the effect of mass included zero (−0.66, 0.00088), whereas when using the other priors, the 95% CIs for this effect did not include zero. Thus, we did not believe our choice of weakly informative priors for the covariate effects and piece-wise hazards significantly impacted our estimates of the posterior distributions.

Statistics describing the posterior distributions of the parameters from the piece-wise constant model are presented in Table 2. It is clear that the morality hazard rate is higher during the first 4 days of life for chicks and drops thereafter based on the significant (i.e., 95% CIs excluding zero), negative parameter estimate for the log hazard effect for ages >4 days. The covariates with significant posterior distributions were chick mass, agricultural nesting habitat, and the 2012 year effect, each of which decreased the mortality hazard rate. These distributions indicate that increasing mass lowers the hazard, chicks in nest sites in agricultural areas had a lower hazard compared to those in grassland habitats, and the hazard was lower in 2012 when compared to 2010. The “unknown” habitat showed weaker effects (i.e., the weight of the posterior distribution was shifted away from zero, but 95% CIs included zero). This effect was positive indicating that there was some evidence in the data that chicks captured in this habitat had an increased hazard compared to grassland nest-site habitats. The remaining covariates appeared to have little influence on the mortality hazard rates. The mean of the posterior for the conditional detection probability (*P*) was 0.75.

**Table 2:** Estimates from the posterior distribution of the parameters (log hazards and log hazard ratios) from the piece-wise constant model for mountain plover chick (*Charadrius montanus*) survival from 2010 to 2012 in Colorado, USA

Parameter	Mean	SD	MC error	0.025 Percentile	0.500 Percentile	0.975 Percentile
Intercept	−2.208	0.253	0.0088	−2.725	−2.202	−1.732
Hazard – Age 5–30 days	−0.479	0.183	0.0040	−0.837	−0.481	−0.114
Chick sex	0.020	0.178	0.0030	−0.333	0.020	0.369
Adult sex	0.077	0.193	0.0043	−0.308	0.077	0.457
Mass	−0.351	0.165	0.0032	−0.667	−0.354	−0.024
Tarsus	0.146	0.175	0.0035	−0.199	0.146	0.488
Prairie dog	0.101	0.188	0.0042	−0.263	0.100	0.467
Agricultural	−0.524	0.267	0.0080	−1.053	−0.523	−0.001
Unknown	0.889	0.455	0.0196	−0.054	0.911	1.712
Year-2011	−0.193	0.202	0.0052	−0.582	−0.195	0.210
Year-2012	−0.548	0.225	0.0057	−0.991	−0.546	−0.111
*P*	0.751	0.019	0.0002	0.713	0.751	0.787
Adult sex impute^1^	0.501	0.028	0.0002	0.460	0.502	0.540
Chick sex impute^1^	0.515	0.031	0.0003	0.455	0.515	0.576

1 Represent probability used to impute missing covariate values.

Our examination of the validity of the proportional hazard assumption did not yield any evidence suggesting violation of the assumption. The posterior distributions of each of the covariate*age interaction terms we examined had their mass centered near zero and each 95% CIs included zero.

## Discussion

Based on our simulation studies, this model performed well across the range of combinations of survival and detection probabilities we examined. The model exhibited good performance even when the proportion of the sample composed of radio-collared individuals was low. However, the RMSE was highest when no radio-collared animals were included in the sample, but this is expected because radio-collared animals provide more information regarding death times compared to marked individuals. The increase in RMSE for the detection probability parameter we observed as the proportion of radio-collared animals in the sample increased is reasonable because this parameter is estimated from the sample of marked animals. Thus, as the number of marked individuals decreases, the available information for estimating the detection probability also declines. In total, our simulation studies demonstrate our approach performs well across a variety of sample sizes, survival, and detection probabilities, which are common in application. But, it is worth noting that for these simulations, we did not investigate the effects of model misspecification, and therefore, our PBS and RMSE values may be optimistic.

Our case study demonstrated real-world value of our integrated approach. It allowed us to investigate the impacts of various covariates on the mortality hazard rates of plover chicks and provides an effective alternative to discrete-time approaches (Dreitz 2009). Additionally, although it was not our final model from which we made inference, we demonstrated how our method can be extended to account for temporal/spatial correlation in hazard rates through regularization (e.g., random walk model).

Our biological findings are similar to previous studies on chick survival. We observed larger mass is correlated with lower mortality hazard rates, which mirrors Ruthrauff and Mccaffery (2005) who noted that size at hatching influences survival rates of shorebirds. We also found no effect of chick or tending adult sex on the mortality hazard rates, which is in concurrence with Dreitz (2009). Similarly, we found habitat type impacts the mortality hazard rate (Dreitz 2009); however, the hazard rate was lowest for nesting in agricultural habitat, while the remaining habitats had higher associated hazards. This contrasts earlier work that demonstrated nesting habitat located in prairie dog-inhabited grasslands led to the highest survival rates (Dreitz 2009); however, this difference is undoubtedly due to high temporal variability in chick survival associated with annual differences in quality of the habitats to support chicks. This species prefers disturbed areas containing exposed bare ground (Dreitz 2009), which increases in agricultural fields during years with low winter/spring precipitation and presumably increases chick survival as observed in our case study. In contrast, during years with high winter/spring precipitation, agricultural fields may result in lower survival due to limited bare ground and increased crop production. Lastly, the lower hazard in 2012 compared to the previous years is likely a result of reduced seasonal rainfall and extreme precipitation events (e.g., hailstorms) during the chick period.

The high detection probability for our case study suggests that despite failed/weak radio transmitters in the sample, if the chick is still alive, it was detected with a high probability after a malfunction or nondetection event. Thus, the effort to locate animals’ postradio failure appears adequate.

Although we believe our integrated survival model to be unique, other modeling techniques with similar aspects have been proposed in the literature. For example, Bunck et al. (1995) also developed an approach to estimate survival when relocation of radio-marked individuals is uncertain. Their technique employs a modified Kaplan–Meier estimator that utilizes for each survey occasion a potentially unique risk set that only includes individuals detected during that occasion. In contrast, our approach jointly estimates the detection and survival process eliminating the need for varying risk sets and permits learning about the detection process. Additionally, our approach allows greater flexibility with regard to the types of marks that can be deployed (e.g., only radiocollaring a subset of the total marked sample), potentially resulting in substantial economic savings.

Similarly, Conn et al. 2012 and Ergon et al. 2009 proposed frequentist models that share commonalities with our integrated survival model. Conn et al. 2012 examined methods to account for imperfect detection when estimating the force of infection in wild populations. Likewise, Ergon et al. 2009 employed event-time approaches within a multistate framework to estimate the latent distribution of age/time of reproduction while accounting for both capture probabilities and censoring associated with natural mortality. Although similar in some respects to these techniques, our model is unique in the ability to account for multiple data sources that contain a mixture of known and unknown fates when estimating hazard rates. Lastly, an interesting observation is if we choose to use the piece-wise constant hazard function in our model, it essentially can be characterized as a continuous-time Cormack–Jolly–Seber (CJS) model (Pollock et al. 1990) where survival is modeled via a log–log link.

In conclusion, the integrated survival model we have described removes some of the current limitations when using event-time analyses. Our simulation efforts and case study demonstrate that the technique performs well and has real-world applications. The strength of this modeling approach is that it can be used for a wide array of survival estimation problems where the fates of all individuals are not known with certainty. This may arise from different marking or follow-up techniques, where some individuals are not detected with certainty or as in our mountain plover case study when nondetection arises from equipment failures. We believe that our integrated modeling approach provides researchers greater access to the powerful machinery of event-time analyses, permitting the realistic modeling of the dynamic unfolding nature of these processes in time, and facilitating the expanded use of these cost-effective tools in arenas not previously possible.

## Data accessibility

The data and R code used are available as online supporting material.

## Supporting Information

**Data S1.** R code used in for the simulation studies.

**Data S2.** Field data used in the case study analysis.
