# Supplementary material for: Integrated survival analysis using an event-time approach in a Bayesian framework
Source: Ecol Evol. 2015 Jan 17;5(3):769–80. doi: 10.1002/ece3.1399 (PMC4328778; doi:10.1002/ece3.1399)
Supplement: Supplementary file 1 [file ece30005-0769-sd1.docx]

**Integrated survival analysis using an event time approach in a Bayesian framework**

**Supporting Information**

**Daniel P. Walsh^1,^^[[1]](#footnote-1)^* Victoria J. Dreitz^2^, and Dennis M. Heisey^1^**

*^1^National Wildlife Health Center, United States Geological Survey, 6006 Schroeder Road, Madison, WI 53711, ^2^Wildlife Biology Program and Avian Science Center, College of Forestry and Conservation, University of Montana, MT 59812*

The following R code was used in the simulation studies described in the manuscript. We present this code with no guarantees to accuracy or limitations associated with its use. We have annotated the code throughout to improve its clarity. This code was modified for the case study in the manuscript. Current research efforts are improving the speed of the code and increasing its generality by taking advantage of C++ capabilities via Rcpp and RcppArmadillo packages available in CRAN.

###Necessary Packages

library(LaplacesDemon) #No longer available on CRAN, but available @ http://www.bayesian-inference.com/index

###Necessary Functions

###Following functions create contributions to the likelihood for use Model statement of LaplacesDemon

nevseen<- function(rin2,ein2,J,cstimes,cstimesa,numzero,test,indices1){

einsuba<-ein2[rin2==0]

stimesa<-diff(cstimesa)

inter<-findInterval(einsuba,cstimesa)

qqq1a<-matrix(0,length(stimesa)+2,numzero)

for(i in 1:length(einsuba)){

val<-vector("numeric",nrow(qqq1a))

if(einsuba[i]%in%cstimesa){einsuba[i]<-0;inter[i]<-inter[i]-1;

val1<-c(rep(0,inter[i]+2),seq(1,(J+2-inter[i]-2),1));val[-indices1]<-val1;

qqq1a[,i]<-val}else{ ##can't be observed on day captured

einsuba[i]<-cstimesa[inter[i]+1]-einsuba[i]; val1<-c(rep(0,inter[i]+1),seq(1,(J+2-inter[i]-1),1));

val[-indices1]<-val1;qqq1a[,i]<-val} #assign zero prob observation to change points if no survey occurs then

}

qqq2a<-array(0,dim=c(length(stimesa)+2,length(stimesa)+2,numzero)) ###create array of survival times (upper triangle)

ccc<-c(0,0,as.numeric(stimesa))

for(i in 1:(length(stimesa)+2)){

qqq2a[1:i,i,1]<-ccc[1:i] #creates upper triangular matrix

}

if(numzero>1){

qqq2a[,,2:numzero]<-qqq2a[,,1]

}

for(i in 1:numzero){

for(j in 2:(inter[i]+2)){ #note +2 because first row is zero for combining with failures

if(j==inter[i]+2){qqq2a[j,j:(length(stimesa)+2),i]<-einsuba[i];break}else{

qqq2a[j,j:(length(stimesa)+2),i]<-0;qqq2a[,j,i]<-0;qqq2a[j,,i]<-0}

}

}

qqq3a<-matrix(0,length(stimesa)+2,sum(rin2==0))

for(i in 1:numzero){

for(j in 1:(inter[i]+1)){

if(j==inter[i]+1){qqq3a[j,i]<-einsuba[i];qqq3a[(j+1):(nrow(qqq3a)-1),i]<-as.numeric(stimesa[j:length(stimesa)]);

break}else{

qqq3a[j,i]<-0;qqq3a[j,i]<-0;qqq3a[j,i]<-0}

}

}

####Those never seen

qqq11a<-matrix(qqq1a,1,)

qqq22a<-matrix(qqq2a,length(stimesa)+2,)

pvec1<-as.vector(sapply(indices1,function(x,y){z<-x+(seq(0,ncol(y)-1,1)*nrow(y))

return(z)},qqq3a))

return(list(qqq11a,qqq22a,qqq3a,pvec1))

}

ll3bfunc<-function(lamtest,lamtest2,a,b,b1,p,indices1,pvec1){

qqq33a<-as.matrix(-lamtest2*b1) #use lamtest2 here

qqq33a[indices1-1,]<-qqq33a[indices1,]+qqq33a[indices1-1,] ##correct failure for lambda change points in failures

if(is.matrix(qqq33a)){qqq33a[nrow(qqq33a),]<-NA}else{

qqq33a[length(qqq33a)]<-NA

}

qqq33la<-matrix(qqq33a,1,) #converts to vector

bbba<-exp(crossprod(-c(1,lamtest),b)) #add 1 to lambda vector because of leading zero added for first failure

ddda<-1-exp(qqq33la) #failures

lasta<-which(is.na(ddda))

ddda[lasta]<-1 #set last row to 1 for calcuation since prob 1 of dying after end of surveys

eeea<-(1-p)^a #detections

eeea[pvec1]<-0 #set lambda change points=to zero so don't contribute to likelihood

out1a<-eeea*bbba*ddda

out2a<-matrix(out1a,nrow(b),)

ll3b<-colSums(out2a)

return(ll3b)

}

#############################################

reps<-500

output1<-matrix(0,reps,12) #Matrix to hold simulation results

colnames(output1)<-c("beta","Lb-beta","Ub-beta","lambda1","lb-lam1","ub-lam1","lambda2","lb-lam2","ub-lam2",

"p","lb-p","ub-p")

for(jjj in 1:reps){

set.seed(jjj)

n<-80

nradios<-60 #number of radios in marked sample

near<-n-nradios #number of eartags/marks in sample

####################Generate the data

surv<-0.55 #true overall annual survival

psurvey<-0.4 #true detection probability of eartagged animals during each survey

M<-2 #number of different hazards(hunting, non-hunting)

mbegin<-"2012-9-1" #begin of marking

sbegin<-"2012-9-16" #begin of surveys

mend<-"2012-9-30" #end of marking

sdate<-"2012-10-1" #start of first hazard

e1date<-"2013-1-1" #end of first hazard (hunting)

e2date<-"2013-10-1" #end of second hazard (non-hunting)

end<- "2013-12-22" #end of surveys

t1<-as.numeric(difftime(as.Date(sdate),as.Date(mbegin)))

t2<-as.numeric(difftime(as.Date(e1date),as.Date(sdate)))

t3<-as.numeric(difftime(as.Date(e2date),as.Date(e1date)))

t4<-as.numeric(difftime(as.Date(end),as.Date(e2date)))

tin<-c(t1,t2,t3,t4)

hazratio<-c(1,2,1,2) #relative to non-hunting hazard (i.e, lambda-hunt/lambda-no hunt)

lambda1<--log(surv)/(t3+hazratio[2]*t2) ##annual survival = surv (Note:t2+t3=365)

lambdas<-c(lambda1,hazratio[2]*lambda1,lambda1,hazratio[2]*lambda1)

######Generate entry times

eday<-round(runif(n,0,as.numeric(difftime(mend,mbegin))))

edate<-eday+as.Date(mbegin) #rounds it to day

##Generate failure/death times

prob1<-matrix(0,n,length(lambdas)+1)

prob1[,1]<-pexp(tin[1]-eday,rate=lambdas[1])

for(i in 1:nrow(prob1)){

prob1[i,2:length(lambdas)]<-pexp(tin[2:length(lambdas)],rate=lambdas[2:length(lambdas)])

}

prob<-matrix(0,n,length(lambdas)+1)

prob[,1]<-prob1[,1]

deathint<-rep(0,n)

for(i in 1:nrow(prob)){

for(j in 2:(ncol(prob)-1)){

prob[i,j]<-prob1[i,j]*cumprod(1-prob1[i,])[j-1]

}

prob[i,ncol(prob)]<-1-sum(prob[i,])

deathint[i]<-which(rmultinom(1,1,prob[i,])>0)

}

T1<-rep(0,n)

for(i in 1:n){

if(deathint[i]==max(deathint)){T1[i]<-NA;next} #if right censored

ifelse(deathint[i]!=1,T1[i]<-rtrunc(1,"exp",0,tin[deathint[i]],rate=lambdas[deathint[i]])+cumsum(tin)[deathint[i]-1]-eday[i],

T1[i]<-rtrunc(1,"exp",0,tin[1]-eday[i],rate=lambdas[1]))

}

T1e<-T1+as.Date(edate)

sday<-7 #number of days between surveys

ndays<-as.numeric(difftime(as.Date(end),as.Date(mbegin)))

numsurvey<-floor(ndays/sday)

svydate<-as.Date(sbegin)+sday*c(0:numsurvey) #survey times from begin of surveys

svydate<-svydate[svydate<=as.Date(end)] #constrain surveys into study period

J<-length(svydate)-1 #number of survey intervals NOT # of surveys

stimes<-difftime(svydate[-1],svydate[-length(svydate)])

###Establish first known dead and last known alive for radio-marked animals (assumes no reporting of dead...telemetry only known dead)

###NOTE:Adjust for staggered entry in the for loop

##s - length of time from last known alive to first known dead

##rin1 - length of time from marking to last known alive

s<-vector("numeric",length(T1e)) #note using T1e=failure+entry times because survey times are based on study start

rin1<-s

for(i in 1:n){

if(is.na(T1[i])){s[i]<-NA;rin1[i]<-difftime(svydate[J+1],edate[i]);next} #If alive past end of surveys

index<-findInterval(c(edate[i],T1e[i]),svydate)

if(index[1]==index[2]){s[i]<-difftime(svydate[index[2]+1],edate[i]);rin1[i]<-0;next} #If die before next survey

rin1[i]<-difftime(svydate[index[2]],edate[i])

s[i]<-difftime(svydate[index[2]+1],edate[i])

}

###Sample marked animals

rmarked<-c(1:nradios)

#radiomark data

ein1<-eday[rmarked] #entry time

rin1<-rin1[rmarked] #last known alive, adjusted for entry time

sin1<-s[rmarked] #first known dead time, adjusted for entry time

####create time vectors for amount of time spent under various hazards

########################################################################Radiocollars

test<-c(0,cumsum(tin))

t1r<-matrix(0,length(lambdas),nradios) #for entry time to last known alive

t1s<-matrix(0,length(lambdas),nradios) #for last known alive to first known dead

###time for entry to last known alive

for(i in 1:nradios){

if(is.na(sin1[i])){t1r[,i]<-tin;t1r[1,i]<-t1r[1,i]-ein1[i];next} #right censored

int1<-findInterval(c(ein1[i],(rin1[i]+ein1[i])),test) #need to add in entry time because intervals are from origin

if(int1[1]==int1[2]){t1r[int1[1],i]<-rin1[i];next} #if enter and last seen in same interval

if(int1[1]<int1[2]){

t1r[int1[1],i]<-tin[int1[1]]-ein1[i] #interval of entry

for(j in (int1[1]+1):int1[2]){ #intervals between entry and last known alive

if(j<int1[2]){t1r[j,i]<-tin[j]} else{ #if known alive during intervals time=interval length

t1r[j,i]<-rin1[i]+ein1[i]-test[j] #if last known alive occurs in interval (need add in entry time)

}

}

}

}

###time for last known alive to first known dead

for(i in 1:nradios){

if(is.na(sin1[i])){next} #t1s<-all zeros...no contribution to likelihood

int2<-findInterval(c(round(rin1[i]+ein1[i]),round(sin1[i]+ein1[i])),test,rightmost.closed=TRUE)

if(int2[1]==int2[2]){t1s[int2[1],i]<-sin1[i]-rin1[i];next} #if last seen and confirmed dead in same interval

if(int2[1]<int2[2] ){

t1s[int2[1],i]<-test[int2[1]+1]-(rin1[i]+ein1[i]) #interval of last known alive

for(j in (int2[1]+1):int2[2]){ #intervals between entry and last known alive

if(j<int2[2]){t1s[j,i]<-tin[j]} else{ #if known alive during intervals time=interval length

t1s[j,i]<-sin1[i]+ein1[i]-test[j] #if last known alive occurs in interval (need add in entry time)

}

}

}

}

###########BINOMIAL OBSERVATION MODEL

emarked<-c((nradios+1):n)

#eartag data

ein2<-eday[emarked] #entry time

eint<-findInterval(edate,svydate) #find entry interval, correct num surveys available if marked after start of surveys

T1eint<-findInterval(T1e,svydate)

###Create interarrival times of observation only during surveys-binomial process (i.e, when observed)

r<-rep(0,n)

aa<-matrix(0,near,J+1)

ii<-0

for(i in emarked){

ii<-ii+1

if(is.na(T1e[i])){aa[ii,(eint[i]+1):(J+1)]<-rbinom((J+1-eint[i]),1,psurvey)}

if(!is.na(T1e[i]) & T1eint[i]>eint[i]){aa[ii,(eint[i]+1):T1eint[i]]<-rbinom((T1eint[i]-eint[i]),1,psurvey)}

if(sum(aa[ii,]>0)){r[i]<-max(which(aa[ii,]==1))}

else{r[i]<-0}

}

nsight2<-rowSums(aa)

#redo for binomial

rin2<-r[emarked] #last known alive survey occasion

eint<-eint[emarked]

t2r<-matrix(0,length(lambdas),near) #for entry time to last known alive

cstimes<-c(0,cumsum(as.numeric(stimes)))

cstimes<-cstimes+as.numeric(difftime(sbegin,mbegin)) #time of surveys from start of marking/origin

rin2t<-sapply(rin2,function(x,y){out<-ifelse(x==0,0,y[x])

return(out)},cstimes)

for(i in 1:near){

if(rin2[i]==(J+1)){t2r[,i]<-tin;t2r[1,i]<-tin[1]-ein2[i];next} #observed on last occasion

if(rin2t[i]==0){t2r[,i]<-0;next} #if never observed does not contribute to survival likelihood portion

int3<-findInterval(c(ein2[i],rin2t[i]),test) #do not need to add in entry time because rin2t=survey time last seen

if(int3[1]==int3[2]){t2r[int3[1],i]<-rin2t[i]-ein2[i];next} #if enter and last seen in same interval

if(int3[1]<int3[2]){

t2r[int3[1],i]<-tin[int3[1]]-ein2[i] #interval of entry

for(j in (int3[1]+1):int3[2]){ #intervals between entry and last known alive

if(j<int3[2]){t2r[j,i]<-tin[j]} else{ #if known alive during intervals time=interval length

t2r[j,i]<-rin2t[i]-test[j] #if last known alive occurs in interval (need add in entry time)

}

}

}

}

#############Create matrices for last term (unseen) in binomial observation model

cstimesa<-c(cstimes,test[-1])[order(c(cstimes,test[c(-1,-length(test))]))] ##include change points

survint<-findInterval(cstimesa,test,rightmost.closed=TRUE)

survint2<-mapply(function(x,y){if(x%in%test){y<-y-1}

return(y)},x=cstimesa[-length(cstimesa)],y=survint[-length(survint)]) #adjust for changes in lambda

survint<-c(survint2,survint[length(survint)])

####Animals seen >0 times

#################VECTOR INPUT – Improve speed in LaplacesDemon

rin2sub<-rin2[(rin2!=0 & rin2<J+1)] #remove those observed on last survey (go into dbinom only) and those never observed

ein2sub<-ein2[(rin2!=0 & rin2<J+1)]

indic.svy<-cstimesa%in%cstimes

indices<-which(indic.svy==FALSE) #where lambda change points occur

indices1<-indices+1

qqq1<-matrix(0,length(cstimesa),length(rin2sub))

for(i in 1:length(rin2sub)){

vala<-vector("numeric",nrow(qqq1))

val1<-c(rep(0,rin2sub[i]),seq(1,(J+1-rin2sub[i]),1))

vala[-indices]<-val1

qqq1[,i]<-vala ###create matrix of exponents for (1-p) ranging from zero to end-last known alive

}

rin2sub2<-sapply(rin2sub,function(x,y,z){return(x+sum(y[-1]<z[x]))},test,cstimes) #adjust last known alive for change point inclusion

qqq2<-array(0,dim=c(length(cstimesa),length(cstimesa),length(rin2sub))) ###create array of survival times

stimes2<-matrix(diff(cstimesa),length(rin2sub),length(diff(cstimesa)),byrow=TRUE)

stimes2<-cbind(0,stimes2)

for(i in 1:length(rin2sub)){

k<-0

for(j in (rin2sub2[i]+1):nrow(qqq2)){

qqq2[(rin2sub2[i]+1):(rin2sub2[i]+k+1),j,i]<-as.numeric(stimes2[i,(rin2sub2[i]+1):(rin2sub2[i]+k+1)])

k<-k+1

}

}

qqq3<-matrix(0,length(cstimesa),length(rin2sub))

for(i in 1:length(rin2sub)){

qqq3[rin2sub2[i]:(nrow(qqq3)-1),i]<-stimes2[i,(rin2sub2[i]+1):ncol(stimes2)] ###create array of failure times

##staggered one back from survival times so (1-s) corresponds to next interval, first entry only (1-s), last will be set =1

#because prob 1 of failing after surveys end=1

}

###for those never seen

numzero<-sum(rin2==0)

if(numzero==0){results<-list(NA,NA,NA,NA)} ##set to missing if all animals seen >0 times

if(numzero!=0){results<-nevseen(rin2,ein2,J,cstimes,cstimesa,numzero,test,indices1)}

###Following creates one large vector from matrices and arrays for faster calculation in Laplaces Demon

##########Seen at least once

qqq11<-matrix(qqq1,1,)

qqq22<-matrix(qqq2,length(cstimesa),)

qqq3[nrow(qqq3),]<-NA #set to NA so can set last row to 1 in ddd

pvec2<-as.vector(sapply(indices,function(x,y){z<-x+(seq(0,ncol(y)-1,1)*nrow(y))

return(z)},qqq3))

##############Estimation from Generated Data

########################Binomial Resighting Model

n1<-nradios #number of radios

n2<-near #number of eartags

nsight2<-nsight2

Navail<-ifelse(rin2==0,NA,rin2-eint)

mon.names <- c("LP","lambda1","lambda2","p")

parm.names <- as.parm.names(list(log.lambda1=0,beta=0, logit.p=0))

PGF <- function(Data) return(c(runif(1,-10,0),rnorm(0,sqrt(5)),runif(1,-10,0)))

MyData <- list(n1=n1, n2=n2, t1r=t1r, t1s=t1s,t2r=t2r, r2=rin2, eint=eint, indices1=indices1,indices=indices,

pvec1=results[[4]],nsight=nsight2, Navail=Navail, survint=survint, qqq11=qqq11,qqq22=qqq22,qqq3=qqq3,

pvec2=pvec2,qqq11a=results[[1]],qqq22a=results[[2]],qqq3a=results[[3]],J=J+1, PGF=PGF,

mon.names=mon.names, parm.names=parm.names)

####DEFINITION OF DATA INPUTS

#t1r - radios last known alive (time broken up by hazard intervals)

#t1s - radios first known dead (time broken up by hazard intervals)

#t2r - eartags last known alive (time broken up by hazard intervals)

#t1s2 - eartags time from last known alive to end of survey (time broken up by hazard intervals)

#rin2 - number of the last survey eartag observed

#eint - number of surveys before eartag entry time

#nsight2 - the number of times eartag observed

#survint - hazard interval containing surveys

#J - number of survey interval +1 = total number of surveys

#indices/indices1 - location of hazard change points for animals seen >1 time|never seen,respectively

#qqq11 - vector for exponent of (1-p) for animals not observed on last survey

#qqq22 - matrix for survival based on survey times for animals not observed on last survey

#qqq3 - vector for failure based on survey times for animals not observed on last survey

#qqq11a - vector for exponent of (1-p) for animals never observed during surveys

#qqq22a - matrix for survival based on survey times never observed during surveys

#qqq3a - vector for failure based on survey times never observed during surveys

#ind - indicator of animals for vectors

#Navail - Number of surveys known to be available for sighting

Model <- function(parm, Data)

{

### Parameters

log.lam1<-parm[1]

beta<-parm[2]

logit.p<-parm[3]

lambda1 <-exp(parm[1])

lambda2<-lambda1*exp(beta) #log-linear proportional hazards model

p<-invlogit(parm[3])

### Log(Prior Densities)

lambda1.prior <- dunif(lambda1, 0, 1, log=TRUE)

beta.prior<-dunif(beta,0,5,log=TRUE)

p.prior<-dunif(p, 0, 1,log=TRUE)

### Log-Likelihood

lamvec<-c(lambda1,lambda2,lambda1,lambda2) #constant hazard for each interval

lamtest<-lamvec[Data$survint]

lamtest2<-c(lamtest,0) #account for staggering one up of failures for easy multiplication below

#radiocollar contribution - survival

in1<-1-exp(crossprod(-lamvec,Data$t1s)) ###last known alive and first known dead occur in same interval

in1<-in1[in1>0]

LL1<-sum(crossprod(-lamvec,Data$t1r),log(in1))

#eartag contribution - survival

LL2<-sum(crossprod(-lamvec,Data$t2r))

#eartag contribution - observation

#animals observed once

qqq33<--lamtest2[-1]*Data$qqq3 #remove first parameter because can't fail in first interval (all animals here seen >=1x)

qqq33[Data$indices-1,]<-qqq33[Data$indices,]+qqq33[Data$indices-1,] ##correct for lambda change points in failure times

qqq33l<-matrix(qqq33,1,)

bbb<-exp(crossprod(-lamtest,Data$qqq22)) #survival portion

ddd<-1-exp(qqq33l) #failure portion

last<-which(is.na(ddd))

ddd[last]<-1 #set last row to 1 for calcuation since prob 1 of dying after end of surveys

eee<-(1-p)^Data$qqq11

eee[Data$pvec2]<-0 #set change points to zero

out1<-eee*bbb*ddd #last binomial term

out2<-matrix(out1,nrow(Data$qqq22))

ll3a<-colSums(out2)

#animals never observed

if(!is.na(Data$qqq11a) && !is.na(Data$qqq22a) && !is.na(Data$qqq3a)){

ll3b<-ll3bfunc(lamtest,lamtest2,Data$qqq11a,Data$qqq22a,Data$qqq3a,p,Data$indices1,Data$pvec1)}else{

ll3b<-1 ###set to one if all animals seen at least once

}

LL3<-sum(dbinom(Data$nsight[Data$r2!=0],Data$Navail[Data$r2!=0],p,log=TRUE),log(ll3a),log(ll3b))

LL <- sum(LL1,LL2,LL3) #Total Log-Likelihood

### Log-Posterior

LP <- LL + lambda1.prior+beta.prior+p.prior

Modelout <- list(LP=LP, Dev=-2*LL, Monitor=c(LP,lambda1,lambda2,p), yhat=0, parm=parm)

return(Modelout)

}

#Set seed and generate Initial Values

set.seed(jjj*pi) #seed seed for each simulation rep

Initial.Values <- c(runif(1,-10,-3),runif(1,0,2),runif(1,-5,5)) #set Initial Values for parmeters

#Run optimization/MCMC

Fit <- LaplacesDemon(Model, Data=MyData, Initial.Values, Covar=NULL, Iterations=50000, Status=50000, Thinning=1,

Algorithm="HARM", Specs=NULL) ##Note: editing LaplacesDemon function to eliminate the initial checks makes ##code run much faster

if(is.na(sum(Fit$Summary2))){next}

if(Fit$Summary2[2,1]<0){break}

output1[jjj,1]<-Fit$Summary2[2,1]

output1[jjj,2]<-Fit$Summary2[2,5]

output1[jjj,3]<-Fit$Summary2[2,7]

output1[jjj,4]<-Fit$Summary2[6,1]

output1[jjj,5]<-Fit$Summary2[6,5]

output1[jjj,6]<-Fit$Summary2[6,7]

output1[jjj,7]<-Fit$Summary2[7,1]

output1[jjj,8]<-Fit$Summary2[7,5]

output1[jjj,9]<-Fit$Summary2[7,7]

output1[jjj,10]<-Fit$Summary2[8,1]

output1[jjj,11]<-Fit$Summary2[8,5]

output1[jjj,12]<-Fit$Summary2[8,7]

cat(jjj)

cat(",")

}

write.table(output1, file="D://results.Rdata", sep=",") #change file name as needed

1. * Correspondence author. E-mail: dwalsh@usgs.gov [↑](#footnote-ref-1)
